# Memory support training and lifestyle modifications to promote healthy aging in persons at risk for Alzheimer's disease: a digital application supported intervention (Brain Boosters)

**DOI:** 10.1186/s12877-023-04574-x

**Published:** 2023-12-21

**Authors:** S. Tomaszewski Farias, J. Fox, H. Dulaney, M. Chan, S. Namboodiri, D. J. Harvey, A. Weakley, S. Rahman, C. Luna, B. F. Beech, L. Campbell, M. Schmitter-Edgecombe

**Affiliations:** 1 Department of Neurology, University of California, Davis, Sacramento, USA; 2 Department of Biostatistics, University of California, Davis, Davis, USA; 3 Department of Psychology, Washington State University, Pullman, USA

**Keywords:** Alzheimer’s disease, Prevention, Multidomain intervention, Cognitive impairment, Dementia, Subjective cognitive decline, Lifestyle, Memory support, Rehabilitation, Behavioral intervention, Dementia prevention, Subjective cognitive concern, Protocol

## Abstract

**Background:**

Evidence-based interventions to protect against cognitive decline among older adults at risk for Alzheimer’s disease and related dementias (ADRD) are urgently needed. Rehabilitation approaches to support memory and behavioral/lifestyle interventions are recognized as promising strategies for preserving or improving cognitive health, although few previous interventions have combined both approaches. This paper describes the protocol of the Brain Boosters intervention, which synergistically combines training in compensatory and healthy lifestyle behaviors and supports implementation and tracking of new behaviors with a digital application.

**Methods:**

The study utilizes a single-site, single-blinded, randomized controlled design to compare a structured lifestyle and compensatory aid intervention to an education-only self-guided intervention. We plan to enroll 225 community-dwelling adults (25% from underrepresented groups) aged 65 + who endorse subjective cognitive decline (SCD) and low baseline levels of healthy lifestyle behaviors. Both interventions will be administered in group format, consisting of 15 two-hour classes that occur weekly for ten weeks and taper to bi-monthly and monthly, for an intervention duration of 6 months. Participants in both interventions will receive education about a variety of memory support strategies and healthy lifestyle behaviors, focusing on physical and cognitive activity and stress management. The structured intervention will also receive support in adopting new behaviors and tracking set goals aided by the Electronic Memory and Management Aid (EMMA) digital application. Primary outcomes include global cognition (composite of memory, attention, and executive function tests) and everyday function (Everyday Cognition Questionnaire). Data will be collected at baseline and outcome visits, at approximately 6, 12, and 18 months. Qualitative interviews, self-report surveys (e.g., indicators of self-determination, health literacy) and EMMA data metrics will also be used to identify what components of the intervention are most effective and for whom they work.

**Discussion:**

Successful project completion will provide valuable information about how individuals with SCD respond to a compensation and preventative lifestyle intervention assisted by a digital application, including an understanding of factors that may impact outcomes, treatment uptake, and adherence. The work will also inform development, scaling, and personalization of future interventions that can delay disability in individuals at risk for ADRD.

**Trial Registration:**

ClinicalTrials.gov. (NCT05027789, posted 8/30/2021).

## Background

### Rationale

Alzheimer’s Disease and Related Disorders (ADRDs) represents an emerging public health crisis. Prevalence currently exceeds 6 million affected individuals in the U.S. and that figure is predicted to double in the next several decades [1]. In the absence of effective medical treatment, there is a critical need for behavioral interventions to prevent or delay symptom onset. It is now estimated that up to forty percent of dementia may be attributable to modifiable risks (including physical inactivity, depression, low education/lack of cognitive stimulation, among others [2]). As such, reducing these risks may substantially lower dementia prevalence. To this end, a number of randomized controlled trials (RCTs) provide evidence that physical exercise improves cognitive performance in those with impairment [3] and in those at risk for ADRD [4]. Various exercise trials have also found decreased rates of brain volume loss or evidence of other changes in biomarkers [5–10]. Similarly, epidemiological studies show an association between greater engagement in cognitively stimulating activities and slower cognitive decline and enhanced neuroplasticity [11, 12]. Recently, randomized intervention trials aimed at enhancing cognitive stimulation, such as learning a new skill [13, 14] or volunteering, [15, 16] have been associated with enhanced cognition and markers of brain structure and function [17, 18]. Better management of stress and depression have also been associated with various aspects of health [19] including enhanced cognitive and brain health [20–23]. Relatedly, there is growing support for the impact of mindfulness and positive psychology-based approaches to enhance emotional well-being and brain health [24–28]. While targeting a single risk factor has some advantages, multidomain preventative interventions that target multiple risks, could have enhanced efficacy and/or be applicable to more individuals than single-component interventions [29, 30]. Trials such the Finnish Geriatric Intervention Study to Prevent Cognitive Impairment and Disability (FINGER) demonstrate that a multidomain intervention related to lifestyle modifications can have a positive impact on reducing dementia risk [29].

Despite the growing interest in multidomain treatment trials, memory support/compensation training has generally not been incorporated into such interventions and this represents a major gap in dementia risk reduction strategies. Providing rehabilitation-based training in compensatory aids, such as memory notebooks and calendars, use of ‘to do’ lists to accomplish daily tasks as well as longer-term goals, and the implementation of organizational strategies has been shown to improve or maintain everyday cognition and functional independence in older adults with neurodegenerative disorders [31–33]. Such training is considered a standard of care for treating individuals with cognitive deficits associated with traumatic brain injury and stroke [19, 34]. Combining training in lifestyle modification and memory support strategies may confer synergistic benefits. Skill enhancement in developing and tracking goals using a calendar and daily ‘to do’ list will likely facilitate the uptake and maintenance of health behaviors. Further, use of digitally-based compensatory tools co-located within one application (app) has many advantages, including providing: 1) a suite of tools that can be used in combination, 2) automated reminder prompts, 3) search tools to help locate information when needed and 4) analytic tracking functions that can help individuals monitor change in health behaviors and provide graphic feedback and enhance motivation.

Given mounting evidence that the pathological changes associated with ADRD begin decades before symptom onset [35], initiating preventative strategies as early as possible is imperative. One approach is to target older adults at high risk for developing ADRD but who are asymptomatic. While targeting those with positive biomarkers may be ideal, it is currently cost prohibitive on a large scale. The presence of subjective cognitive decline (SCD) represents a significant risk for future cognitive decline and ADRD [36, 37]. Many individuals with SCD harbor other indicators of early disease (e.g., amyloid [3, 4, 24], brain atrophy [5, 24]). Thus, the period in which SCD is present but cognition remains broadly normal offers a critical window of opportunity to intervene to build resilience by developing compensation strategies to use in everyday life and to reduce modifiable risks for decline and dementia. Further, because cognitive functions remain intact at this stage, the ability to learn new skills and behaviors and develop long-term habits is enhanced. In fact, multiple reviews/meta-analyses have concluded that behavioral interventions in individuals with SCD are associated with positive cognitive benefits, especially when incorporating cognitive training/cognitive rehabilitative training as in the current RCT [38–40].

### Study objectives

The overall objective of this study is to test, in a RCT, a multidomain intervention combining training in rehabilitation-based memory support strategies and lifestyle/health behavior modification on cognitive and functional outcomes. To capitalize on a critical window of opportunity to intervene, we will target cognitively normal older adults with SCD, an established ADRD risk. Two groups will be compared. The target intervention (referred to as the structured group) combines training in memory support strategies and healthy lifestyle modification and sets specific behavioral target goals that participants are encouraged to meet and actively track. It makes use of a senior-friendly Electronic Memory and Management Aid (EMMA) digital application [41, 42] to both build and support memory compensation strategies, and to support self-monitoring of healthy behavior goals, an important component in promoting health behavior change [43]. The structured intervention is theoretically grounded in Self Determination Theory (SDT [44, 45]), with an emphasis on building competency at a stage when cognitive functions remain intact, supporting autonomy through flexibility in choice of lifestyle changes (e.g., what type of physical exercise in which to engage), and support through a group-based intervention. This intervention will be compared to an education only group (referred to as the self-guided group) which provides similar information on compensation strategies and healthy lifestyles, but without providing explicit behavioral goals or support on how to implement the information**.** To meet the study objective, the following aims are planned: 1) evaluate intervention efficacy on primary outcomes including global measures of cognition and everyday function and secondary outcomes including target behaviors (increased compensation, increased physical and cognitive activity and enhanced stress management) and other important health outcomes (e.g., physical ability); 2) evaluate demographic and other characteristics of those who respond to the intervention to better understand for whom the intervention works; 3) evaluate treatment adherence and identify the components of the structured intervention that impact treatment success using a mixed-method approach that combines quantitative and qualitative data collection.

## Methods

### Study design

**Fig. 1:**
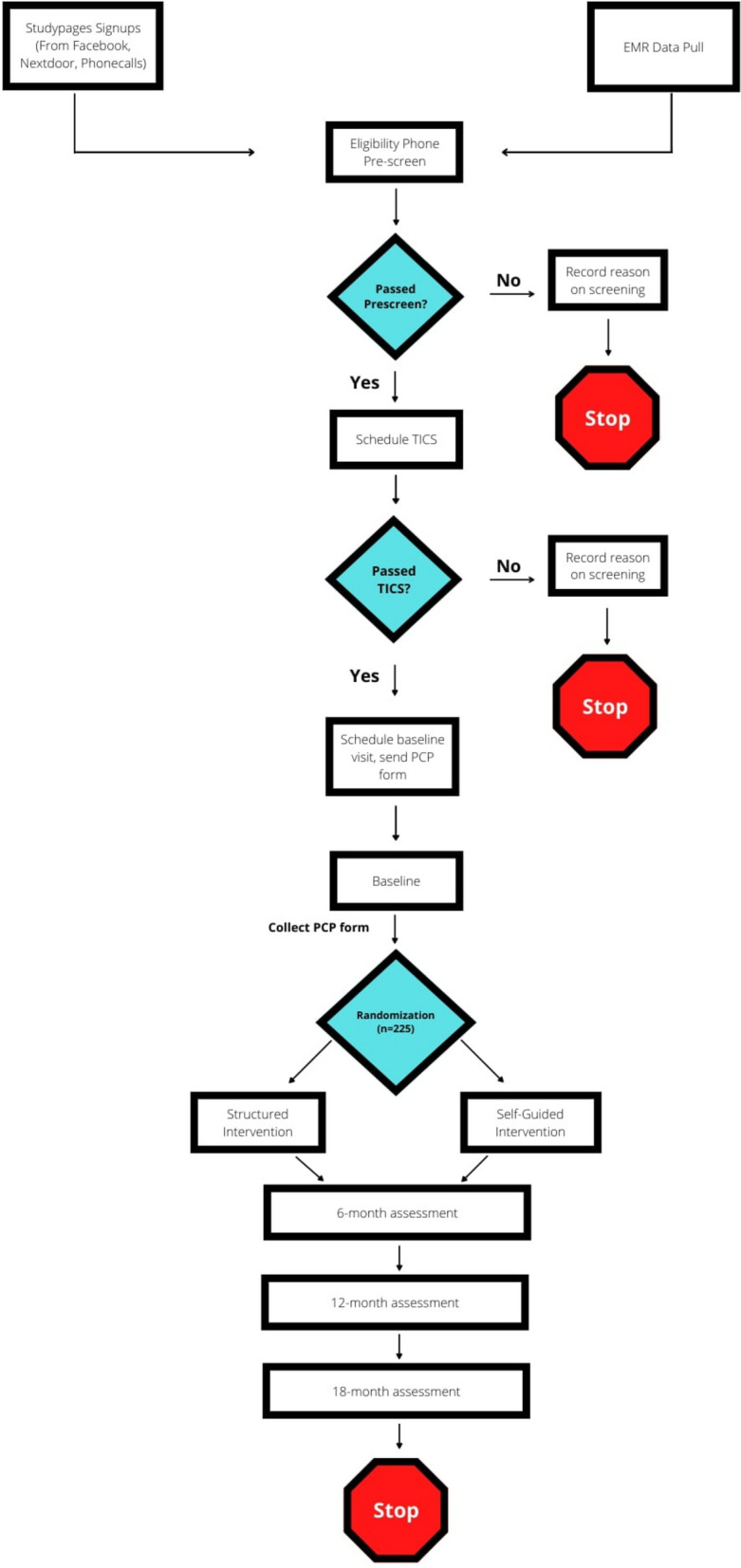
Study flowchart for patient recruitment, intervention administration and data collection

This study utilizes a single-site, single-blinded, randomized controlled design comparing the structured compensation training and lifestyle intervention to an education only (self-guided) intervention. All participant screening and recruitment, intervention administration, and data collection will occur at UCD. The study flowchart is illustrated in Fig. 1. Outcome measures will be collected at 4 timepoints: 1) prior to the intervention (baseline), 2) immediately after the 6-month intervention is complete, 3) 6 months post intervention, and 4) 12 months post intervention. All study staff collecting outcome measures will be blind to the participant’s intervention arm status.

All study procedures are reviewed and approved by Institutional Review Boards (IRBs) at the University of California, Davis (UCD), and Washington State University (WSU) IRB has a reliance agreement on the protocol approved by the UCD IRB. The study is registered on ClinicalTrials.gov. (NCT05027789). All study participants provide written, informed consent before participating in assessments or intervention activities.

### Eligibility and recruitment

The target population for the study is community-dwelling English-speaking individuals age 65 years or older who have SCD. SCD is defined by endorsement of experiencing “a change in your memory or other aspect of thinking in the last 1–3 years” in the context of normal performance on cognitive screening (measured by the Telephone Interview for Cognitive Status (TICS) [46]) and independence in instrumental activities of daily living (IADLS; measured using the Lawton and Brody IADL instrument [47]). Eligibility for the study also includes a relatively low level of engagement in healthy lifestyle behaviors (defined as not engaging in exercise resulting in sweating or elevated heart rate ≥ 3 times per week). Eligible participants must also be willing to utilize an iPad and digital app. Participants are required to obtain approval from their primary care physician to participate in the study to ensure they are sufficiently healthy to engage in physical exercise. Exclusion criteria include a known diagnosis of dementia or cognitive impairment, a known diagnosis of another neurologic disorder that could affect cognition (e.g., Parkinson’s disease), or a history of a major psychiatric disorder (e.g., schizophrenia) or active severe depression or suicidal ideation (as measured by the Patient Health Questionnaire – 9; PHQ-9 [48]).

Multiple recruitment strategies will be utilized to outreach to potential study participants. These strategies will include advertising on social media, attending community-based events (e.g., health fairs), and solicitation through information delivered via the University of California, Davis Health Care System electronic medical records (EMR) system.

### Interventions

#### Overview

As noted above, participants take part in one of two possible intervention arms: the structured or self-guided intervention group. The self-guided education-only intervention was chosen as a comparison group to account for the impact of two general factors: 1) the provision of similar health information, and 2) the social support and stimulation provided by attending a group-based intervention. Each intervention group is matched in terms of the number and length of group sessions and exposure to other group participants and the interventionists. Both interventions are administered in a group format and consist of a total of 15 two-hour classes. The first 10 classes occur weekly, then the classes transition to bimonthly for one month and then monthly for the last three months. This format was chosen to facilitate mastery and gradually evolving autonomy and self-direction. The target size of each class is about 15 people to balance optimal group discussions with individualized attention. All intervention sessions will be digitally recorded. Recordings will be emailed to participants if they miss a session and make-up sessions are provided where needed.

#### Target intervention (Structured Group)

Skill building in memory support strategies focuses on three areas: 1) regular use of a calendar, 2) goal setting and use of task lists, and 3) organizational strategies. Goals of calendar use include supporting better planning and reducing reliance on spontaneous recall. Emphasis is placed on having an active/engaged lifestyle, hence the impetus for scheduling events in the calendar. A key component of this training is to make a *habit* of: a) reviewing the calendar routinely (at designated times daily) and b) entering *all* recurring and non-recurring activities. Training in goal setting and use of a task list encompasses use of a daily ‘to do’ list on which participants place daily tasks they need to complete. Items on the ‘to do’ list are checked off once completed or moved to another day if not completed. Identifying and working towards personalized long-term goals is also a portion of this training module, which includes planning and prioritizing steps to accomplish goals, breaking goals into small steps, and transferring smaller steps to one’s daily ‘to do’ list. The emphasis on long-term goals helps to motivate participants and is driven, in part, by research showing a sense of purpose is important to well-being and health [49]. Organizational strategies focus on structuring one’s environment to maximize the ability to efficiently accomplish daily tasks and to provide external memory cues (such as always placing one’s wallet and keys in a bowl next to the door). Each participant identifies various ‘functional zones’ within their own living environment associated with specific daily activities with which they regularly engage. Functional zones range from one’s home office or desk, garage, kitchen pantry, or even a gym bag (e.g., ensuring it has all essential items such as shoes, socks, relevant equipment and is consistently located in one place). The goal is to enhance the organization of the ‘space’, minimize clutter or unnecessary materials within the space, and ensure critical components are easily available and within close proximity. Organizational strategies for the digital world are also covered with an emphasis on how to organize one’s digital apps and files and how these tools can be used as memory aids (such as creating a digital folder with all of one’s recipes for easy access).

With regard to the healthy lifestyles, we focus on three areas: 1) increasing physical activity, 2) increasing engagement in cognitively and socially stimulating activities, and 3) engagement in activities to aid in stress management and promote a sense of well-being. For physical exercise, the intervention target goal is for participants to engage in at least 150 min of moderately vigorous physical activity (PA) per week. This target was chosen based on recommendations of several well recognized health guidelines [50]. Participants can choose the type, frequency, and duration of PA throughout the week and are encouraged to gradually increase PA intensity/duration over time as their fitness allows. Participants are taught how to gauge the intensity of physical exercise based on standard criteria [51]. This and all components of the intervention are specifically designed based on established behavior change standards. For example, participants are encouraged to develop habits and routines associated with PA (and the other health behaviors promoted in the intervention), identify anticipated barriers, and develop approaches to overcome those barriers. Goal setting, tracking, and monitoring behavior change are also key components of the program. For the intervention component on increasing engagement in cognitively and socially stimulating activities, although no well-established recommendations exist, based on previous work which found the greatest cognitive benefit for those with activity counts of ≥ 12 times per week [52] for at least 20 min at a time, this was set as the intervention goal. Class discussions related to this topic include identifying potential activities (a list is provided but participants are encouraged to generate additional options); there is also a focus on identifying enjoyable activities and engaging in a variety of activities. For the stress management component of the healthy lifestyles, four specific sets of skills based on previous interventions in mindfulness and positive psychology are taught including mindful meditation (including focusing on the breadth and the body scan), developing a gratitude practice with a journaling component, noticing daily positive events, and engaging in acts of kindness. The intervention prescribed goal is engagement in ≥ 3 of any of the stress management techniques per week.

Each participant in the structured intervention arm is provided with an iPad that contains the EMMA app to use throughout the intervention and the 12-month post-intervention follow-up period. Structured intervention sessions incorporate teaching participants to use the EMMA app to support new compensatory strategies, as well as self-monitor, and integrate new lifestyle behaviors into their everyday lives. Each of the compensatory training components are supported by specific EMMA app functions including: a Calendar, a daily To Do List, and a notes section to support goal setting and monitoring and implementation of organizational skills taught in the intervention. The EMMA app contains a section to write about all four of the stress management exercises, and participants create gratitude journal entries in this section. Participants use the EMMA app to plan out and schedule brain health activities. When a health activity is checked as completed, participants are asked to provide either minutes of physical activity or counts for cognitive or stress management activities, enabling them to monitor weekly progress towards the intervention goals. The EMMA app provides participants with graphs of their weekly progress towards the intervention goal for each of the brain health activities and rewards participants when they reach the goal. Use of this digital app also allows for the target intervention components of checking a calendar and to do list, expanding on organizational strategies and functional zones, and tracking health activities to be captured via the EMMA app data metrics.

#### Education self-guided group

The self-guided group receives similar educational information about memory support strategies and healthy lifestyles as the structured group. Participants in the self-guided group are encouraged to utilize this information to develop an individualized healthy lifestyle program that best meets their own goals and schedules. No specific digital app is provided to the self-guided group, although information about commercially available apps aimed at memory support and engagement in healthy lifestyles are discussed where applicable.

### Randomization

After completion of baseline data collection, participants are randomly assigned using a block randomization strategy. Twice as many participants will be randomized into the target intervention to allow us to power Aim 3 (primarily within group analysis). Block sizes of 3 or 6 will be randomly chosen and assignments balanced within each block to ensure balance over time. Randomization is revealed to participants by a nonblinded study coordinator.

### Assessment of intervention fidelity and adherence

Intervention fidelity will be assessed by an independent rater to ensure the presentation of class material is consistent with the content of the intervention manuals. A minimum of 20% of recorded intervention sessions will be reviewed using standardized content checklists. Interventionists will be provided with support and feedback to assure command of the material. Prior work indicates this method produces a high fidelity [53]. The primary measure of adherence to the intervention is number of classes attended (inclusive of make-up sessions and/or view of recorded sessions). For the structured group, participants are encouraged to use the EMMA app daily to manage everyday activities through the calendar, to-do list and notes sections and to track progress with their healthy lifestyle goals. The EMMA app automatically collects data metrics as participants interact with the app, which allows information about participant use of this compensatory tool and progress towards healthy lifestyle goals to be gathered in real-time. Specificity of the EMMA app data metrics allows for analysis of global app usage (e.g., number of daily app uses) as well as for analyses of each individual target intervention component (e.g., total minutes of physical activity entered, note taking to plan an organizational strategy).

### Outcome assessment procedures

#### Primary outcomes

Objectives for the intervention are to improve or maintain cognition and everyday function and as such, these are our primary outcomes (Table 1). Cognitive function is measured by a modified Neuropsychological Test Battery (mNTB), a comprehensive battery of cognitive tests shown to have good psychometric properties [54]. This test battery has been used in a number of intervention trials including both pharmaceutical trials and other behavioral interventions (e.g., U.S. POINTER trial). Use of this battery in the current study will facilitate comparison of our findings with those of other intervention trials aimed at dementia risk reduction. A global cognitive composite score constructed as an average of the z-scores of individual cognitive test scores, will be used as the primary cognitive endpoint. Our primary functional outcome is the Everyday Cognition questionnaire, which was chosen because it was specifically designed to measure very early functional changes [55] and has been shown to be sensitive to preclinical disease [17].

**Table 1:** Primary outcome measures

*Primary Outcomes*	*Specific Measures*
Cognitive Testing: Global Cognitive Composite [54]	Story Recall (memory test) List learning test (memory test) Digit span (attention) Trail Making (executive function) Digit Symbol (executive function) Verbal fluency (executive function) Prospective Memory Test (memory)^a^
Everyday Function	Everyday Cognition (ECog) Questionnaire (self and informant-report versions)^b^ [51]

^a^Not included in the derived composite scores

^b^This questionnaire will also be completed by a study partner to collect additional information about how the participant is functioning in their daily life when possible (not required)

#### Secondary outcomes

Secondary outcomes are listed in Table 2. They focus primarily on behavior changes and skill acquisition related to specific aspects of the intervention program. Because of its potential relevance to many of the memory support strategies taught in the intervention, a prospective memory test was added to the cognitive outcomes.

**Table 2:** Secondary outcome measures

*Secondary Outcomes*	*Specific Measures*
Self-efficacy	Coping Self efficacy (CSES) [56]
Mood and Well-being	Center for Epidemiologic Studies Depression Scale (CES-D) [57] Perceived Stress Scale (PSS) [58] Life Satisfaction [59, 60] Purpose in Life [61] Brief Resiliency Scale [62]
Physical Performance	The Short Physical Performance Battery (SPPB) [63]
Self-rated Health and Quality of Life (QoL)	Patient Reported Outcomes Measurement Information System (PROMIS) General health Quality of Life (QoL) [64]
Instrumental Activities of Daily Life (IADL) Disability	Cooperative Study ADL Prevention Instrument* (ADCS-ADL-PI) [65]
Compensation Strategy Use	Everyday Compensation (EComp)* [66, 67]
Health Behaviors Directly Targeted in the Intervention	Community Healthy Activities Model Program for Seniors (CHAMPS) [68] Positive Affect and Negative Affect Scale (PANAS) [69] Gratitude Questionnaire [70] Mindfulness Questionnaire [71]

^*^ These questionnaires will also be completed by a study partner to collect additional information about how the participant is functioning in their daily life if possible (not required)

### Predictors of treatment response

In accordance with previous intervention research recommendations [72], person-specific characteristics of treatment response are examined. In addition to demographic factors, potential predictors include questionnaires assessing indicators of self-determination (autonomy, competence, and relatedness) and health literacy. The SDT construct of autonomy and self-regulation of health behaviors is measured using the Treatment Self-Regulation Questionnaire (TSRQ) (adapted) [73]. The construct of competence is measured by the Perceived Competence for Healthy Brain Aging (adapted) [74], and the construct of relatedness and self-perceived social support by the Social Support for Health Behaviors (adapted) [75]. Health literacy and the perceived ability to manage one’s health is measured using the Patient Activation Measure (PAM-13) [76]. The following demographic characteristics are also collected: age, sex, level of education, ethnicity, and socioeconomic status (using a 9-point scale from 1 [$0 – $9,999] to 9 [$80,000 and more]).

### Qualitative interviews

Mixed methods research [77] is a valuable way to examine complex health interventions that are built on multiple components that may act both independently and interdependently [78]. To expand understanding of how to deliver a preventative compensation and lifestyle intervention to individuals with SCD, a subset of participants in the structured intervention condition will be asked to complete a qualitative semi-structured interview in which they answer open-ended questions about their experience in the intervention. The goal of the qualitative interviews is to identify barriers and facilitators to uptake of and adherence to the components of the intervention, as well as to elicit feedback that could lead to improvements in the intervention (e.g., components the participants found helpful or unhelpful). This information will complement the quantitative analyses and provide further insight on the relationship between adherence and the primary and secondary outcome measures, components of the intervention important for treatment effectiveness, and reasons for poor uptake. The qualitative interview will be recorded, transcribed, and coded using standard qualitative research methods. We aim to collect this information after completion of the 6-month intervention, as well as at other follow up time points across participants with high and low adherence rates derived from EMMA usage metrics.

### Data and safety monitoring

This study will be monitored by an external Safety Officer (SO), which will act in an advisory capacity to the National Institute on Aging (NIA) and the primary investigators (PIs) to monitor participant safety, data quality, and the progress of the study. The SO will meet with the contact PI (STF) on a quarterly basis and will complete a safety monitoring report to the NIA twice yearly.

We expect adverse events (AEs) associated with this intervention to be minimal and consistent with risks associated with normal daily activities and an active lifestyle (e.g., muscle soreness from physical activity). AEs will be tracked in the study database. We will employ multiple approaches to ensure complete and accurate collection of information about AEs. All participants will be prompted to report AEs and serious adverse events (SAEs) at their outcome assessment visits (approximately 6, 12, and 18 months). Participants may also spontaneously report AEs/SAEs during their intervention meetings. All AEs/SAEs will be reviewed within 24 hours by the study clinician/medical monitor to determine seriousness, whether the event was unexpected and related to the intervention. AEs that are serious, unexpected, and related to the intervention will be reported immediately to the project PIs and to the Institutional Review Board (IRB), SO, and NIA.

### Sample size and power calculations

The goal is to enroll 225 individuals into this trial, anticipating 20% drop-out. The trial was powered to detect intervention effectiveness (Aim 1). Assuming 180 participants complete the entire study with a correlation between repeat assessments of at least 0.4, alpha = 0.05 and a two-sided test, we will have over 80% power to detect a difference in slopes between baseline and immediately post-intervention as small as 0.5 standard deviations (SD). If alpha is reduced to 0.025 to account for the two primary outcomes, the minimum detectable difference in slopes is 0.54 SD. Our preliminary studies had a difference in slopes of 0.5–0.6 SD, although we expect the outcomes proposed for this study to be more sensitive to change in behaviors than those used in our preliminary studies.

### Planned statistical analysis

#### Intervention effectiveness

To evaluate the success of randomization, we will first compare between interventions (structured, self-guided) on demographics and other variables using two-sample t-tests (continuous) and chi-square tests (categorical); Wilcoxon rank sum tests or Fisher’s exact tests will be used instead if assumptions are violated. If differences between intervention groups exist, follow-up analyses will include those variables as covariates to ensure any observed intervention effect is not due to other differences between the groups. Primary analyses will be intent-to-treat analyses, that estimate intervention effects based on groups as randomized. Due to the repeat assessments over time across individuals (baseline, immediately after the 6-month intervention, and at 6- and 12-months post-intervention), repeated measures, random effects models will be used to estimate differences in rate of change between the groups at different time points. The main intervention effect will be defined as the difference between groups from baseline to immediately after the 6-month intervention, although later time points will assess maintenance of those effects. These models will incorporate random intercepts and slopes to account for between-person variation in overall level and change over time. All model assumptions will be checked and transformations or repeated measures approaches for non-normal data, such as generalized estimating equation (GEE) approaches will be used if needed. List-wise deletion will be used if < 5% of the data are missing; multiple imputation will be used if > 5% of the data are missing. If data appear to be missing not at random or exceeds > 20% missingness (which we have put procedures in place to prevent), we will conduct sensitivity analyses using Wu-Carrol and Diggle-Kennward approaches that our team has previously applied in RCT trials [79, 80]. Similar analyses will be completed with the secondary outcome measures.

#### Characteristics of treatment responders

Initial analyses will be similar to those described above for Aim 1 only focusing on indicators of SDT (autonomy, competence, relatedness) and health literacy (HL) to assess change over time in these measures and whether those rates of change differ between intervention groups. In the next set of analyses, key outcomes include the primary and secondary outcomes. For SDT/HL measures that show no change over time for either intervention group and for the other characteristics only measured once (demographics), repeated measures, random effects models, will be used to assess how they are associated with change in trial outcomes (interaction between assessment and SDT indicators or other characteristics). Further interactions with intervention group will also be considered to evaluate differences in the association with change between groups. For SDT/HL measures that do show change, we will use an extension of the repeated measures, random effects called simultaneous modeling [81–83] that treats the variables with repeated measurements as outcomes, leading to multiple outcome variables in a single model. Combined, these analyses will allow us to test the hypothesis that certain sub-groups benefit more from the target intervention.

#### Adherence analyses

Overall adherence will be measured by class attendance across the two groups. For all other analyses we will focus on adherence within the structured group, in this case the primary adherence metric will focus on a total adherence metric (e.g., EMMA usage), but secondary analyses will investigate adherence metrics representing the different intervention components as measured in EMMA (e.g., use of the calendar, weekly healthy lifestyle behaviors). We will utilize the adherence metrics collected during the two weeks prior to the post-intervention assessment as a measure of adherence during the intervention, and we will also assess adherence changes over time through the 6- and 12-month post-intervention assessments. For adherence during the intervention, linear regression will be used with the adherence metric as the outcome and participant characteristics or tool use as independent variables. Transformations or non-linear models will be used if suggested by model diagnostics. Repeated measures, random effects models will be used to assess change over time in adherence and whether certain participant characteristics, or tool use at each assessment, are associated with change in adherence. Finally, repeated measures, random effects models will be used to assess the association between adherence and change in Aim 1 primary and secondary outcomes. If we find that adherence changes over time, change in adherence will be considered as a time-varying independent variable in the models to see how it influences trial outcomes at later assessments.

For the qualitative data analysis, participants’ responses to the open-ended interview questions will be coded and analyzed by a team of trained reviewers. Common patterns related to adherence will be identified and resulting themes will be derived iteratively; the frequency of each theme will be tabulated. These analyses will complement quantitative analyses and provide further insight on the relationship between adherence and the primary and secondary outcome measures, components of the intervention important for treatment effectiveness, and reasons for poor uptake.

## Discussion

Multidomain interventions provide a promising strategy for dementia risk reduction [29]. Critical to this endeavor, the current intervention targets older adults at increased dementia risk due to SCD, but who maintain normal cognition and so can maximally benefit from skill building and healthy habit formation. The technology-assisted structured intervention being examined in this RCT synergistically combines both lifestyle modifications and memory compensatory training to support cognitive health as well as promote functional independence. Very few prior multidomain lifestyle interventions have incorporated compensation training and so this trial is poised to make a unique contribution to the development of dementia risk reduction interventions. The EMMA app, which was specifically designed for older adults, aims to both facilitates engagement in the intervention itself (e.g., through daily tracking of lifestyle goals) and allows for real-time tracking and evaluation of adherence to the specific treatment component (e.g., calendar use, physical activity) [84]. This will provide *new information* about strategies that can be used to boost treatment adherence (use of automated, strategically-timed and customizable motivational prompts) as well as new methods for tracking health status changes in the real-world environment. If successful, such real time data metrics may be used to create algorithms that recognize and predict incipient nonadherence to the intervention and capture change in health status. Altogether, the trial is designed to expand our understanding of factors that may bolster or reduce adherence to and outcomes of the intervention, thereby leading to optimization of a scalable intervention that can delay disability in individuals at risk for ADRD and optimize health and wellness as people age. Specifically, s*uccessful completion of Aim 1* will demonstrate that training compensation and lifestyle intervention can improve cognition and everyday function relative to the education-only control group. Secondary outcomes will examine the impact of the intervention on other behavioral targets including well-being, IADLs, physical function, compensation, cognitive domain scores, and specific lifestyle activities. *Successful completion of Aim 2* will improve understanding of constructs that can be targeted in a compensation and lifestyle intervention and contribute knowledge about whom may benefit most from the interventions, which can optimize future programs. *Successful completion of Aim 3* will provide important practical knowledge about the intervention and its components, which will be used to further inform development of a more acceptable, adaptable and scalable compensation and lifestyle intervention designed to delay disability in individuals with SCD.

## Data Availability

Not applicable.
